# Why SIT Works: Normal Function Despite Typical Myofiber Pattern in Situs Inversus Totalis (SIT) Hearts Derived by Shear-induced Myofiber Reorientation

**DOI:** 10.1371/journal.pcbi.1002611

**Published:** 2012-07-26

**Authors:** Marieke Pluijmert, Wilco Kroon, Alessandro C. Rossi, Peter H. M. Bovendeerd, Tammo Delhaas

**Affiliations:** 1 Department of Biomedical Engineering/Cardiovascular Research Institute Maastricht, Maastricht University, Maastricht, The Netherlands; 2 Department of Biomedical Engineering, Eindhoven University of Technology, Eindhoven, The Netherlands; University of California San Diego, United States of America

## Abstract

The left ventricle (LV) of mammals with Situs Solitus (SS, normal organ arrangement) displays hardly any interindividual variation in myofiber pattern and experimentally determined torsion. SS LV myofiber pattern has been suggested to result from adaptive myofiber reorientation, in turn leading to efficient pump and myofiber function. Limited data from the Situs Inversus Totalis (SIT, a complete mirror image of organ anatomy and position) LV demonstrated an essential different myofiber pattern, being normal at the apex but mirrored at the base. Considerable differences in torsion patterns in between human SIT LVs even suggest variation in myofiber pattern among SIT LVs themselves. We addressed whether different myofiber patterns in the SIT LV can be predicted by adaptive myofiber reorientation and whether they yield similar pump and myofiber function as in the SS LV. With a mathematical model of LV mechanics including shear induced myofiber reorientation, we predicted myofiber patterns of one SS and three different SIT LVs. Initial conditions for SIT were based on scarce information on the helix angle. The transverse angle was set to zero. During reorientation, a non-zero transverse angle developed, pump function increased, and myofiber function increased and became more homogeneous. Three continuous SIT structures emerged with a different location of transition between normal and mirrored myofiber orientation pattern. Predicted SIT torsion patterns matched experimentally determined ones. Pump and myofiber function in SIT and SS LVs are similar, despite essential differences in myocardial structure. SS and SIT LV structure and function may originate from same processes of adaptive myofiber reorientation.

## Author Summary

Deciphering the structure-function relation in healthy hearts is important to understand cardiac pathologies. In the structure-function relation, the myofiber orientation patterns play a central role. Between people with normal organ arrangement (Situs Solitus, SS) this pattern is strikingly similar. Such consistency in myocardial structure might be the result of an adaptation process to accommodate for homogeneous distribution of myofiber strain across the wall and for optimal pump function. The heart of people with a mirror-imaged position of their organs (Situs Inversus Totalis, SIT) has a modified myofiber orientation pattern with respect to SS: normal at the LV apex, but mirrored at the base. Hence, studying SIT hearts provides a unique possibility 1) for understanding adaptation mechanisms related to myofiber orientation and mechanical load, and 2) to gain additional insights into the structure-function relations of the LV. Through mathematical modeling of LV mechanics, we have found that myofiber orientation pattern in both SS and SIT may originate from same processes of adaptive myofiber reorientation. After reorientation, pump and local myofiber function were found to be similar between SS and SIT as well: a remarkable finding when considering the large difference in myofiber orientation pattern.

## Introduction

The myofiber orientation pattern in the cardiac left ventricular wall has an invariant nature among mammals, including humans, with a normal organ arrangement (Situs Solitus, SS) [1]–[4]. Myofibers follow a left-handed helical path near the epicardium and gradually change their pitch through a circumferential path in the midventricular wall towards a right-handed helical path near the endocardium. The transmural change in helix angle 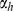 is qualitatively the same from apex to base. Moreover, myofibers cross over between endo- and epicardium. The direction of crossover gradually changes from apex to base [5] and is quantified by the transverse angle 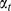.

**Figure 1:**
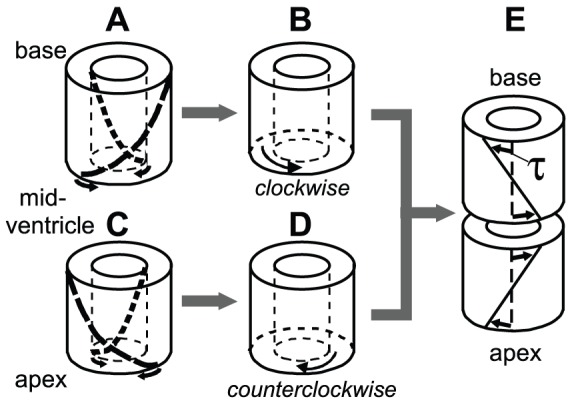
Relation between myofiber orientation and torsion. 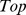
: At the base of the Situs Inversus Totalis (SIT) LV, myofibers follow a right-handed helical path at the sub-epicardium (**A**). Contraction of these myofibers, tends to rotate the midventricle in a clockwise direction with respect to the base, when viewed from the apex. The opposite is true for the sub-endocardium: myofibers follow a left-handed helical path, and contraction of these myofibers tends to rotate the midventricle in a counterclockwise direction with respect to the base (**A**). In general, a net clockwise rotation is measured at the base in SIT LV [6], indicating that epicardial myofibers dominate endocardial myofibers (**B**). 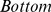: At the apex of the SIT LV, myofibers follow the same pattern as in the Situs Solitus (SS) LV. The dominant myofibers at the sub-epicardium follow a left-handed helical path (**C**). During contraction, a net counterclockwise rotation is measured in the apical region with respect to the midventricle of the SIT LV or with respect to the base in the SS LV (**D**). In fact, **C** and **D** both represent a whole SS LV. Finally, the torsion angle 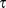 in SIT is similar to SS at the apex and inverted at the base (**E**).

SS LVs not only display an invariant myofiber pattern, but also a large similarity in experimentally determined measures of deformation, such as torsion [6], [7]. Contraction of sub-endocardial myofibers with a right-handed helical orientation tends to rotate the apex in a clockwise direction with respect to the base, when viewed from the apex (figure 1C). The opposite is true for the sub-epicardium: contraction of myofibers with a left-handed helical orientation, tends to rotate the apex in counterclockwise direction during myofiber contraction. A net counterclockwise rotation of the apex as obtained from measurements [6], indicate that epicardial myofibers dominate endocardial myofibers (figure 1D).

Several model studies demonstrated that myofiber orientation pattern is a major determinant of strain distribution in the cardiac wall [7]–[9]. In addition, magnetic resonance tagging (MRT) studies showed that myofiber shortening during ejection exhibits little heterogeneity [10]. Even more so, when coefficients of a polynomial that described the spatial distribution of myofiber orientations were optimized for minimal heterogeneity in myofiber shortening during ejection, realistic myofiber orientations were found [11]. Consequently, it was hypothesized that reorientation is an important adaptive mechanism for a myocyte to achieve a preferred mechanical loading state. Indeed, in a computational model of shear-induced adaptive myofiber reorientation, global LV pump as well as local myofiber function increased upon reorientation, while the latter displayed less spatial heterogeneity [12]. This suggests that the invariant nature of myofiber orientation in the SS LV reflects the unique solution of a successful adaptation process at myocyte level.

Scarce experimental and anatomical studies demonstrated that in individuals with Situs Inversus Totalis (SIT), i.e., a complete mirror image of their organ anatomy and position, the myofiber orientation pattern of the LV is not a complete mirror image of the pattern in the SS LV [6], [13]–[15]. Instead, in SIT LVs the transmural change of 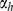 at the apex is as in the SS LV but it changes to a (partially) mirror-imaged transmural distribution at the base [6], [13], [15]. Anatomical data suggest that the transition between the two distributions seems to be located more apically at the endocardium than at the epicardium [13], [15], but detailed information is lacking. In addition, no quantitative data on the transmural course of 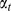 have yet been obtained.

As can be expected considering the dependency of cardiac deformation on myofiber orientation pattern, torsion in the SIT LV was found to differ from that in the SS LV. At the apex, torsion patterns of SS and SIT coincide, whereas at the base an inverted torsion pattern is observed in SIT when compared to SS (figure 1E). More interestingly, torsion patterns have been shown to differ considerably in between SIT LVs [6]. This suggests that myofiber orientations of the SIT LV not only deviate from that in the SS LV, but also display variation among SIT LVs themselves. Assuming that myocytes in the SIT LV have a normal adaptive response through reorientation, this adaptation process seems to result in multiple outcomes. Despite differences in deformation (and structure), none of the subjects in the SIT group studied by Delhaas *et al.* [6] showed any cardiac complaints.

**Figure 2:**
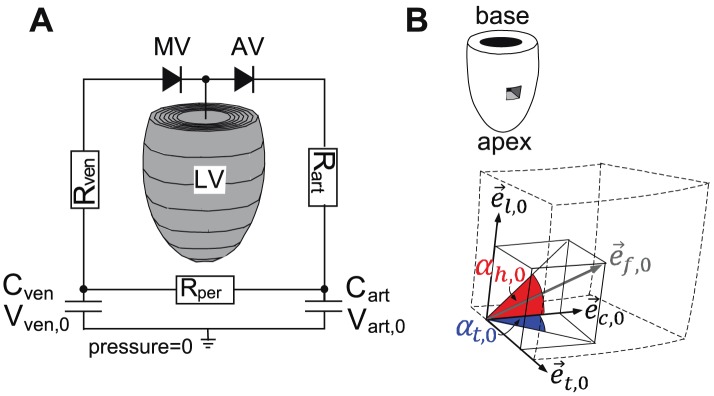
Computational model of LV mechanics. **A**: The ellipsoidally shaped finite element (FE) mesh of the left ventricle (LV) consists of 60 elements and is incorporated in a lumped parameter model of the circulation. AV, aortic valve; 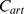, arterial compliance; 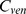, venous compliance; MV, mitral valve; 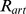, arterial resistance; 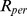, peripheral resistance; 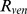, venous resistance; 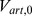, zero-pressure arterial volume; 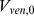, zero-pressure venous volume. **B**: Description of myofiber orientation vector in the unloaded state 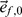 by helix angle 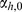 and transverse angle 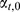 using a local cardiac coordinate system {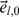, 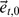, 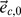}.

**Figure 3:**
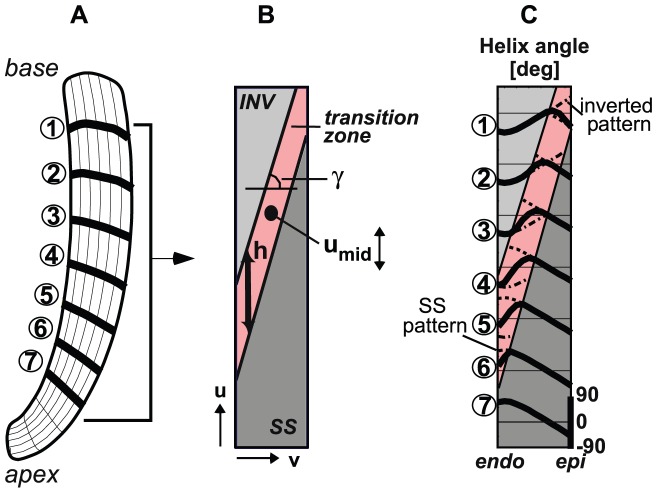
Initial myofiber orientation in SIT simulation *MID*. **A**: Long-axis cross-section of LV mesh. **B**: Mapping of the cross-section in **A** on a rectangular domain used to describe the spatial distribution of myofiber orientations. The transition from a normal (SS) pattern at the apex to an inverted (INV) pattern at the base is described by parameters 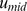 (location of transition at midwall), 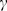 (the slope of the transition between the endo- and epicardium), and 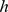 (the height of the transition zone). 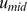 is subject of variation in between the SIT simulations *BASE*, *MID* and *APEX*. **C**: Initial (before reorientation) transmural course of helix angle 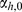 in simulation *MID* (solid) at the 7 levels indicated in **A**. In the transition zone, the courses of the SS (− −) and inverted pattern (−.) are presented. The transmural distribution of the transverse angle 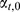 was set to zero.

In this study, we addressed the question whether variations in myofiber patterns of the SIT LV can be predicted by adaptive reorientation of myofibers, and whether these various outcomes yield similar pump and myofiber function as in the SS LV. To investigate this, we employ a mathematical model of LV mechanics [9] (figure 2) and include shear-induced adaptive myofiber reorientation [12]. In the latter model, we assume myofibers to adapt their orientation as a response to local loss of myocardial integrity due to forces generated by fiber cross-fiber shear strains during myofiber contraction. Scarce information on the distribution of 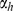 in the SIT LV is used to set a non-zero initial condition for 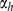 (figure 3) in the adaptation model. We performed three SIT simulations in which the longitudinal location of the transition between the normal and inverted transmural distribution of 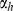 is varied. The transition is located halfway between base and apex in simulation *MID*, more towards the base in simulation *BASE*, and more towards the apex in simulation *APEX*. It is expected that variation in this location might explain the inter-individual differences in torsion in SIT. In absence of experimental data, the initial condition for 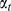 was set to zero. For reference purposes, a situs solitus simulation *SS* was performed in which a normal initial distribution of 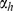 was set [11]. As adaptation proceeded, we analyzed local and global LV function and compared model computed torsion with experimental torsion data.

## Results

**Figure 4:**
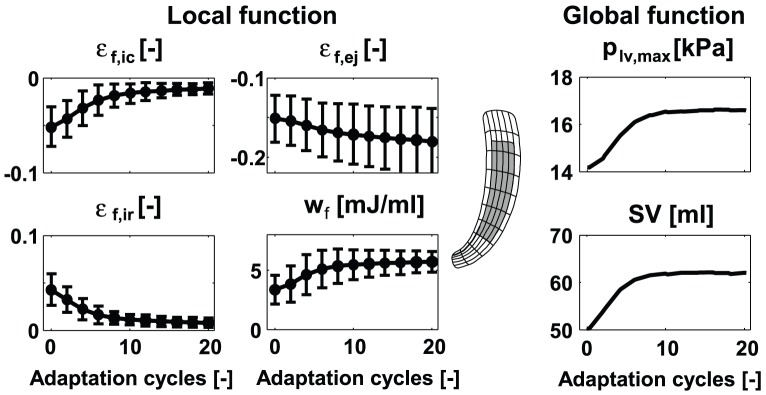
Evolution of local (left) and global (right) LV function in SIT simulation *MID* during the reorientation process. Local function is presented by means and standard deviations (SD) of variables natural myofiber strain during isovolumic contraction 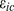, during ejection 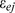, during isovolumic relaxation 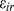, and stroke work density 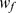. The values were calculated from the grey area indicated in the long-axis cross-section of the LV mesh (mid). Global function is presented by maximum LV pressure 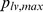 and stroke volume 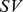.

In all simulations, local and global LV function increased significantly during the adaptation process as indicated by the increase in 1) myofiber shortening (decrease of myofiber strain) during ejection 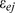, 2) stroke work density 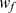 (area enclosed by myofiber Cauchy stress-natural myofiber strain loop), 3) maximum left ventricular pressure 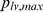, and 4) stroke volume 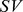. In addition, fiber strains during the isovolumic contraction (IC) and relaxation (IR) phases, 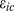 and 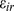, decreased significantly as a result of minimizing fiber cross-fiber shear. As an example, evolution of local and global function in simulation *MID* is shown in figure 4. Parameter values all reached a steady state value after about 15 adaptation cycles. In the steady state, standard deviations (SD) of the local function parameters are significantly decreased, which indicates increase in homogeneity. For example, the SD of 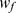 decreased with 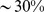.

**Figure 5:**
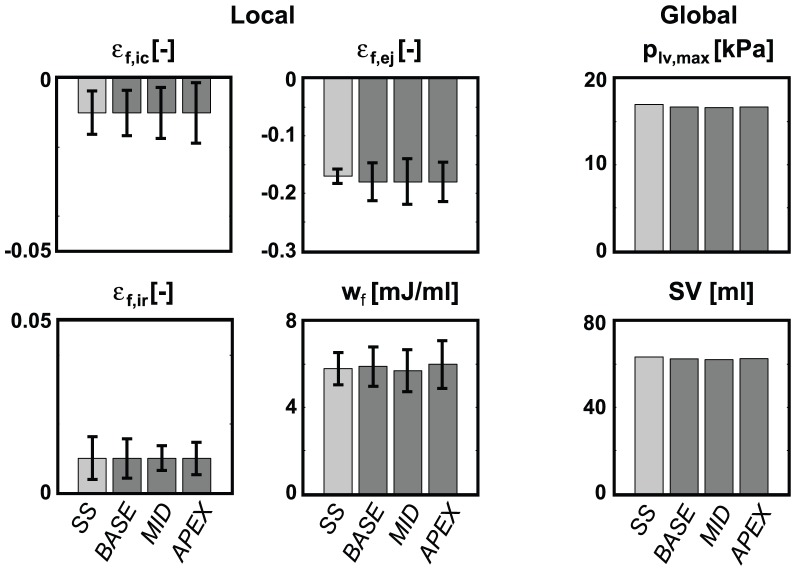
Final LV function after 15 adaptation cycles in the SS simulation and the three SIT simulations (*BASE*, *MID*, *APEX*). Mean values and standard deviation (SD) of local function parameters are presented left, values of global parameters right. Differences between simulations are not significant.

After 15 adaptation cycles, function parameter values are not significantly different between the SIT simulations. Neither are the values in the SIT simulations significantly different in comparison to the SS simulation (figure 5). In simulation *SS*, 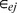 exhibits less heterogeneity when compared to the SIT simulations.

**Figure 6:**
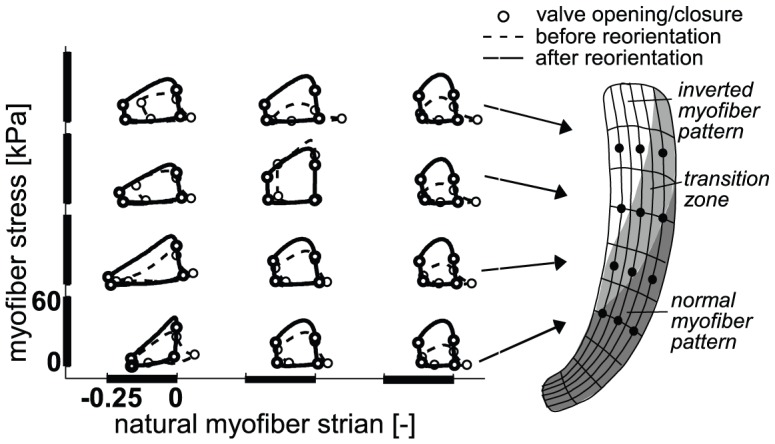
Change in local mechanics as a consequence of myofiber reorientation in SIT simulation *MID*. Myofiber Cauchy stress-natural strain loops are analyzed in the nodes indicated in the LV mesh (right) before (− −) and after (−) reorientation. The location of the transition zone before reorientation is also indicated in the LV mesh.

Local myocardial function in simulation *MID* is shown in more detail in figure 6. Myofiber Cauchy stress-natural strain loops are shown before (dashed line) and after 15 adaptation cycles (solid line) in several nodes across the LV wall. After reorientation, the loops become more homogeneous, as was also indicated by the decrease in SD of myofiber strains and stroke work density (figure 4). Although homogeneity increased significantly, locations in or near the transition zone in the SIT LV still show deviating local myocardial function after reorientation. This results in, for example, a larger SD in 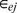 when compared to simulation *SS* (figure 5).

**Figure 7:**
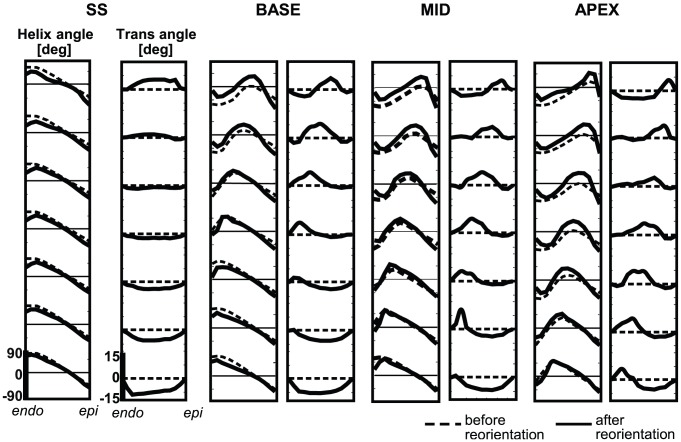
Transmural course in myofiber angles before (− −) and after (−) myofiber reorientation. Results of simulation *SS* (left), and SIT simulations *BASE*, *MID* and *APEX* (right) are shown. Analysis is done at 7 different levels in the LV wall (see figure 3A).

Figure 7 shows transmural distributions of the helix angle 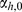 and transverse angle 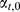 in the mechanically unloaded state, indicated by subscript 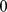 (see figure 2B). Results are shown before (dashed line) and after 15 adaptation cycles (solid line) at 7 levels between apex and base. In all simulations, relatively small changes are observed between initial and final distributions of 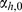. Though 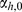 changed, especially in the transition zone, transmural patterns stayed present.

Larger changes are observed between initial and final distributions of 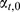. In simulation SS, 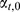 develops a characteristic pattern from positive basal values to negative apical values at midwall. In the SIT simulations, the pattern is more complex. In simulation *MID*, 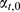 at the basal level varies from negative values at the endocardium to positive values at the epicardium. Going from base to apex, the region of positive 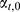 shifts towards the endocardium and a region of negative 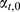 develops near the epicardium. In simulations *BASE* and *APEX* the pattern is similar, except for a shift towards base and apex, respectively.

**Figure 8:**
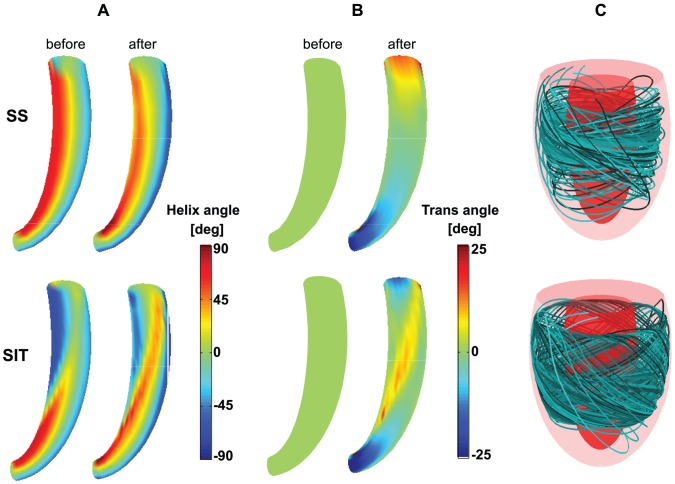
Structural results of simulation *SS* (top row) and SIT simulation *MID* (bottom row). **A**: Helix angle 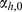 distribution on a long axis cross-section of the LV mesh before (left) and after (right) reorientation. **B**: Transverse angle 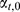 distribution before (left) and after (right) reorientation. **C**: 3-D visualization of fiber paths through the LV mesh after reorientation. Ten paths are shown which started at different locations between endo- and epicardium. The color of the path refers to the starting point.

In figure 8, myofiber angles 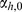 and 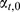 are shown on a long axis cross-section of the LV mesh. Since fiber angles in the SIT simulations are generally the same, results of SIT simulation *MID* are presented next to the results of *SS* to visualize the difference in distribution pattern between SIT and SS. During adaptation, 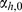 changes more in SIT than in SS, especially in the transition zone. In SS, the transmural course of helix angle 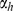 is qualitatively the same from apex-to-base, whereas in SIT the transmural course of 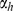 changes from apex-to-base. In SS, the maximum amplitude of 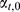 is located near the endocardium and changes from negative at the apex to positive at the base. In SIT, maximum 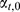 is located near the endocardium at the apex, but shifts towards the epicardium near the base. In addition, largest values of 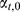 appear in the transition zone. In this respect, all SIT simulations resulted in similar structures. Though, in simulation *BASE*, the area with highest amplitudes of 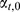 is located more towards the base, and in simulation *APEX* more towards the apex. The translation of fiber angle distributions into a 3-D structure is presented in figure 8C. From 10 different points between endo- and epicardium but at the same level between apex and base, fiber paths were followed resulting in the partially filled LV as shown in the figure.

**Figure 9:**
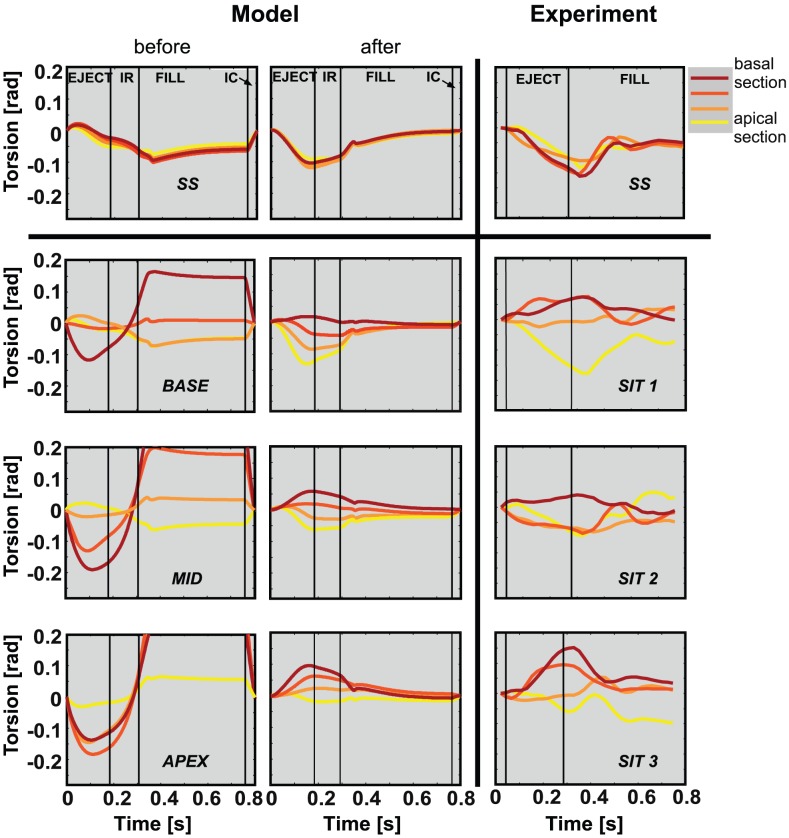
Torsion [rad] during a cardiac cycle. Results are presented from simulation SS (upper left), an SS subject (upper right), 3 SIT simulations (bottom left), and 3 SIT subjects (bottom right). Model results are shown before and after reorientation. Torsion was determined in four sections between apex and base. 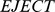 = ejection phase; 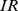 = isovolumic relaxation phase; 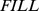 = filling phase; 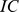 = isovolumic contraction phase.

Figure 9 shows the results of torsion patterns in both model (left) and experiment (right). After reorientation, torsion patterns have changed significantly, especially in the SIT simulations. Torsion amplitudes after reorientation (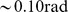) are significantly lower than those before reorientation (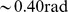) and more in agreement with experimentally observed amplitudes (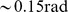). After reorientation, the torsion patterns, which are negative in the SS LV, become less negative in simulation *BASE* and may even invert in simulation *APEX* during ejection. Thus, the torsion patterns shift along with the transition zone. For each of the torsion patterns in the SIT simulations, a corresponding pattern could be found in the experimental data set of 8 SIT subjects in [6]. In simulation *SS*, torsion is homogeneous between the sections, which is observed in all 9 SS subjects in [6] as well.

## Discussion

In this study, different SIT LV structures were estimated using a finite element (FE) model of LV mechanics [9] including shear-induced myofiber reorientation [12]. In comparison to our previous study [16], geometry is more realistic and fibers are allowed to crossover between endo- and epicardium. Fibers reorient as a response to shear instead of shortening during ejection, and no constraints are prescribed for fiber orientation at apex or base, allowing the structure to develop without restrictions. Although model set ups are different, this study also showed that local (myofiber) and global (pump) function in the SIT LV is similar to that in the SS LV. In contrast to the previous study, we now showed the possibility of multiple SIT LV structures and the importance of the transverse angle. The final distributions of the helix angle and transverse angle could be considered as the first detailed suggestion for fiber orientations in SIT.

In figure 8 it was shown that although the final SIT LV structure is essentially different from the final SS LV structure, it is a continuous structure. Fibers followed a path through the whole ventricular wall, as in the SS LV. Although no experimental data is available to confirm the model predicted structures, the similarities in model computed and experimental torsion indicate that the estimated structures might be realistic.

As a consequence of myofiber reorientation, local and global LV function increased significantly in all simulations. This suggests that, as in the SS LV, mechanical work could indeed be distributed homogeneously in the SIT LV too. Moreover, the location of the transition from a normal myofiber orientation pattern at the apex to an inverted pattern at the base had no influence on the local and global SIT LV function. Finally, SIT LV function was comparable to SS LV function, which is in agreement with the finding that SIT individuals display no cardiac complaints [6].

**Figure 10:**
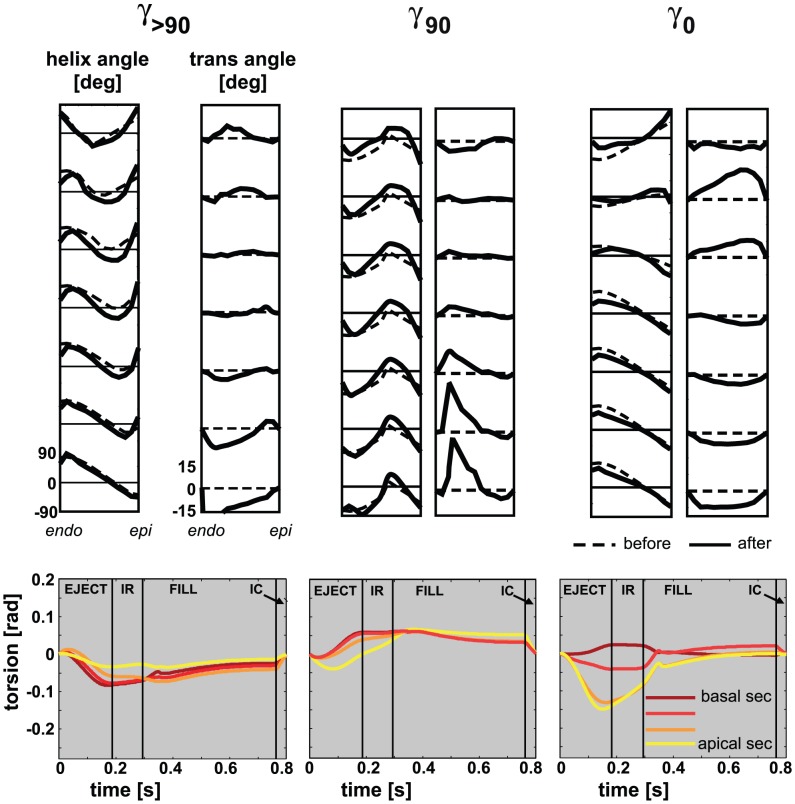
Results of additional simulations. The additional simulations were performed with a variation on simulation *MID* (with 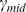, see figure 3 for definition of 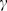): 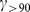 (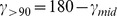), 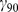, and 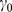, where the subscript 90 and 0 refer to a 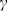 of 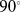 and 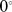, respectively. **Top**: Transmural distribution of 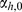 and 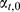 before (− −) and after 15 adaptation cycles (−) at 7 levels between apex and base. Major pattern of 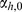 remained closed to the initial distribution, while a non-zero distribution for 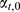 developed. **Bottom**: Torsion patterns [rad] after 15 adaptation cycles. Amplitudes of torsion have decreased significantly after reorientation as compared to amplitudes before reorientation.

The choice of simulations with fixed 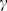 and 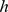, and a variation in 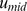 was based upon the scarce available data on myofiber orientation [13], [15] and deduced from experimental findings on torsion [6]. As far as we know, other SIT structures, for example characterized by a substantial variation in 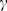, are not reported in literature. Yet, to investigate the space of feasible solutions, we performed additional simulations. These new simulations are a variation on simulation *MID* (with 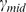): 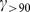 (with 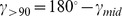), 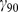, and 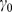, where the subscript 90 and 0 refer to a 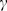 of 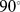 and 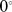, respectively. In all additional simulations, both local and global LV function developed according to the patterns shown in figure 4. In addition, LV function after 15 adaptation cycles was not statistically different from that shown in figure 5. Fiber orientation also developed similarly to the results shown in figure 8: after adaptation, the distribution of 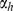 was still close to the initial distribution, while 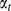 developed a non-zero distribution (see figure 10). Torsion amplitude decreased significantly upon adaptation. Consequently, our model predicts the existence of many fiber architectures, characterized by a case specific match of the distributions of helix and transverse angles.

This finding seems to contradict the finding of our previous study in the SS LV, where we concluded that the effect of the initial condition of the helix angle disappeared upon adaptation [17]. Apparently, in the latter study the initial conditions for 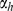 were close enough for the remodeling process to end up in the same solution. In our SIT simulations *BASE*, *MID* and *APEX*, the differences in initial conditions persist after remodeling, although they are limited to a base-to-apex shift of the transition zone only. Out of the many possible solutions predicted by our model, only the SIT structures and the SS structure are found in practice. This suggests that additional physiological mechanisms exist, that regulate myofiber orientation.

### Comparison with experimental data

In figure 9 it is shown that agreement between model computed and experimentally determined torsion is significantly better after reorientation. The agreement suggests that the estimated structures could indeed be realistic. Thus, an abnormal torsion pattern could coincide with normal LV function [18]. The inter-individual differences in torsional deformation could originate from a different location of the transition in LV structure from normal at the apex to inverted at the base.

Because of relaxation of spins in the magnetized tissue, the strength of the MR signal decreases over time. This makes tracking of the tags more difficult at the end of the filling phase. The decrease in reliability of the estimation of torsion towards the end of the cardiac cycle is evident from the non-zero values of torsion amplitude: considering the cyclic deformation of the myocardium, these values are expected to return to zero.

The maximum amplitude of torsion occurred earlier in the model than in the experiment. This observation indicates that the timing of increase and decrease of active stress development in the model is not entirely realistic. However, the difference in timing had no influence on the increase in homogeneity in function nor on the gradient in torsion amplitude, which was developed in the SIT simulations after reorientation.

### Study assumptions and limitations

The results of the shear-induced adaptation may have been influenced by the absence of sheets in the constitutive model of the tissue. Sheets are predominantly oriented in transmural direction, facilitating thickening of the wall [19], [20]. Their effect on normal and shear stiffness of the tissue has been demonstrated in experiments [21] and quantified in constitutive models [22]. Similar to the hypothesis on myofiber reorientation that we used to estimate myofiber orientations, the orientation of these sheets has been linked to shear as well [23]. As such, extension of the adaptation model by including sheets and reorientation thereof could be considered as a next step.

As mentioned before, other adaptation mechanisms are likely to be active as well. Clinically, one of the most evident examples of adaptation is the change in LV wall mass and cavity volume in response to pressure and volume overload, respectively. In addition, in reality the externally unloaded LV exhibits a transmural gradient in sarcomere length with epicardial sarcomeres being longer than endocardial ones [24]. This might be a result of mechanically induced adaptation as well. Extension of the model with these adaptive mechanisms should be considered.

In this study, torsional deformation was used to compare results of model and experiment. We also compared model predictions of the deformation mode circumferential-radial shear to experimental data. Similar to our previous study for SS [25], circumferential-radial shear decreased substantially upon fiber reorientation, but final patterns did not match experimental findings. The discrepancy is mainly explained by the large sensitivity of this shear component to the setting of 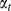 [7]. In addition, the discrepancy suggests that our model of shear-induced remodeling of fiber orientation must be complemented by other remodeling laws.

Our cardiac mechanics model has several limitations. For example, the onset of contraction was assumed to be homogeneous, despite the fact that there is a delay in electrical activation of about 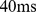. This assumption is motivated by the observation that, at least in the normal healthy heart, a homogeneous onset of contraction yields more realistic strains than assuming the timing of the onset of contraction to follow the electrical activation [26]. LV shape, the major determinants of which are the ratio of cavity to wall volume and eccentricity, was based on data from dog hearts [27], [28]. LV size was set to 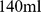, the average volume of the dog hearts used to validate the original model [29]. This volume is representative for a small human heart as well, as indicated by the cardiac output of about 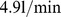 in our simulations. Since tissue mechanics does not depend on absolute size and the influence of shape is minor [30], we consider our description of LV geometry adequate for this study.

Geometry and structure of the LV were assumed rotationally symmetric, while interaction of the LV with the right ventricle (RV) was not taken into account. Myofiber orientations show differences between septum and LV free wall [31] that could originate from the mechanical interaction of LV and RV. If experimental data of myofiber orientations in the SIT LV can be obtained, they should be measured in the free wall, since the effect of interaction will be least for this region. Our predictions on 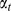 suggest that these experiments might focus on the finding that the region of maximum positive 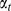 shifts from the epicardium to the endocardium, when traveling from base to apex.

### Conclusions

In this study, we have found that local and global LV function in SIT and SS were similar, despite essential differences in myocardial structure. Using the same processes of shear-induced myofiber reorientation, both SS and SIT LV structures were estimated by this adaptation mechanism and the structures were continuous. The space of feasible solutions predicted by the model turned out to be larger than the experimentally found variation in structures. This suggests that additional physiological mechanisms exist that regulate myofiber orientation. Large agreement in torsion data between model and experiment suggests that measured interindividual differences in torsion pattern could originate from different locations of the transition from a normal myofiber orientation pattern at the apex to an inverted pattern at the base.

## Methods

### Ethics statement

All subjects gave informed consent prior to enrolment in the study, in accordance to the joint ethical committee of Maastricht University and Academic Hospital Maastricht.

### Model of left ventricular mechanics

Tissue deformations during the cardiac cycle are calculated with a generic finite element (FE) model of LV mechanics. With respect to geometry, material properties and the circulation in which the LV is embedded, this FE model is identical to the model presented in [9]. Therefore, it will only be described in brief.

#### Geometry

In the passive stress-free state, a thick-walled geometry is assumed (figure 2A). The endocardial and epicardial surfaces are described by truncated ellipsoids. In this state, wall and cavity volumes equal 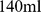 and 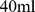, respectively.

#### Material properties

Myocardial tissue Cauchy stress 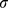 is composed of a passive component 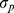 and an active component 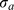:
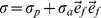
(1)with 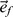 the current myofiber direction in the deformed tissue. Passive material behavior is assumed nonlinearly elastic, transversely isotropic, and nearly incompressible. The mathematical description of the strain energy density function can be found in [9] and is based on experiments in dogs [32].

Active stress 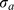 is modeled through a series arrangement of a contractile and a series elastic element. The magnitude of 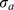 depends on time elapsed since activation 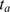, sarcomere length 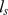, and sarcomere shortening velocity 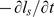 [26]:
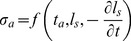
(2)Principles of the active material behavior are based on experiments in dogs [33]. Parameters values for the active material are derived from experiments in rats [34], [35]. Active stress development is initiated simultaneously at each location in the LV wall with a cycle time of 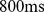.

#### Governing equations and boundary conditions

In the model, the quasi-static equations of conservation of linear momentum are solved:
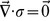
(3)with 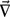 the spatial gradient operator. At the base, essential boundary conditions are defined to suppress rigid body motion and to represent the mechanical effect of structures left out of the model, e.g., the valvular annulus. Axial displacement is suppressed at the whole basal surface, whereas circumferential displacement is suppressed at the endocardial basal ring only. The epicardial surface is assumed to be traction free while the endocardial surface is uniformly subjected to left ventricular pressure 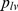. During isovolumic contraction (IC) and relaxation (IR) phases of the cardiac cycle, 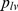 is determined such that mechanical equilibrium of the myocardial tissue is obtained at a constant end-diastolic or end-systolic LV volume, respectively. During the filling and ejection phase, 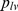 is computed from the interaction of the LV with the circulation according to a lumped parameter model [9] (figure 2A). Parameter values of the circulation model are based on the human hemodynamic system.

#### Numerical implementation

The equilibrium equations (3) are solved numerically with a Galerkin type finite element method using 27-noded hexahedral elements with a tri-quadratic interpolation of the displacement field. Because the model is rotationally symmetric, tissue displacements are described in a right-handed cylindrical coordinate system {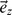, 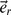, 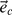} with the axial direction 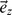 defined from apex-to-base. This allows description of the LV wall with 1 circumferential element, which reduces computational demand significantly. In total, the LV wall is represented by 60 elements: 6 elements in radial, 1 in circumferential and 10 in longitudinal direction.

### Myofiber orientation

The myofiber orientation 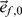 is prescribed with respect to the local cardiac coordinate system {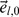, 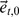, 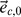} (figure 2B), where the subscript *0* refers to the mechanically unloaded state. The transmural direction 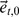 is defined as the outer normal to the cardiac surfaces. The longitudinal direction 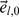 is defined perpendicular to 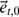 from apex to base. To obtain a right-handed coordinate system, the circumferential direction 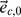 is defined in clockwise direction when viewing the LV in apex-to-base direction. Myofiber orientations are described by two angles. The helix angle 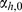 is defined as the angle between 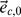 and the projection of 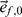 on the circumferential-longitudinal plane (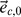, 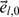). The transverse angle 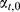 is defined as the angle between 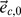 and the projection of 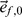 on the circumferential-transmural plane (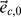, 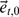).

### Myofiber reorientation

We simulated myofiber reorientation with the model by Kroon *et al.* [12]. In this model, it was assumed that structural changes of myofiber orientation occur as a response to local loss of myocardial integrity due to forces generated by fiber cross-fiber shear strains during myofiber contraction. These shear forces are assumed to damage connections between extra-cellular matrix (ECM) and myofibers. New connections are formed continuously during both the diastolic and systolic phase of the cardiac cycle. When a connection is made, the actual orientation field tends to be fixed within the tissue. This conceptual model was translated into a mathematical model in which the myofiber orientation in the unloaded state 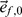 will evolve towards the actual myofiber orientation 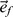 corrected for rigid body rotation. In a previous study, we have shown that this mechanism leads to a realistic myofiber orientation pattern in the SS LV [12]. In particular, a non-zero 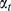 developed, that caused improved correspondence between model predicted and experimentally determined patterns of LV circumferential-radial shear strain and torsion [25].

### Simulations performed

One SS simulation and three SIT simulations were performed. In all simulations, the first 10 consecutive cardiac cycles were used to reach a hemodynamic steady state and myofiber reorientation was not included. In subsequent cycles myofiber reorientation was simulated throughout the whole LV.

#### Initial myofiber orientation in SS

At the start of the adaptation process, the transmural distribution of 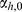 in SS (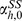) is described using the parameterized distribution in [9]. It varies nonlinearly with the transmural position from endocardium to epicardium (figure 7, dashed lines in left graph). This spatial distribution is a function of normalized coordinates (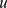, 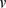, figure 3B). The normalized longitudinal coordinate (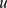) varies linearly with the geodesic distance from the equatorial plane. It changes from 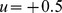 in the basal plane, through 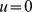 at the equator to 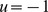 at the apex. The normalized transmural coordinate (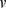) varies linearly with the distance in the ventricular wall from 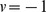 at the endocardial surface to 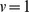 at the epicardial surface. The initial condition for transmural distribution of 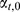 is set to zero.

In both SS and SIT the parameterized description of fiber orientation is abandoned during adaption, and myofiber orientation is adapted per node.

#### Initial myofiber orientation in SIT

According to anatomical data of SIT LVs, the helix angle 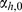 must change from a normal transmural course at the apex to an inverted transmural course at the base [15]. Inverted 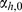 (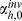) is defined as
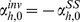
(4)The initial transition from an SS to an inverted pattern is characterized by three parameters: the location at midwall between apex and base 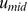, the slope of the transition between the endo- and epicardium 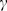, and the height of the transition zone 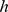 (figure 3B). In the LV region below the transition zone, the transmural course of 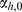 is as in the SS LV (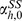). In the region above the transition zone, the transmural course of 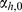 follows an inverted pattern (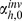). Across the transition zone, 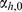 changes linearly from 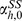 to 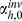 (figure 3B and C). As in simulation *SS*, the initial transmural distribution of 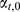 is set to zero.

In this study, the location of the transition zone 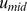 was subject of variation, whereas 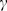 and 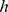 remained unchanged. The transition is located at 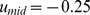 in simulation *MID*, more towards the base (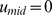) in simulation *BASE*, and more towards the apex (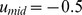) in simulation *APEX*. In the additional simulations (results presented in figure 10), 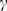 was subject of variation while 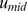 and 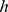 remained unchanged.

### Postprocessing

#### Quantification of LV function

Local function was quantified by changes in mean and standard deviation (SD) of four parameters quantifying mechanical tissue load: stroke work density 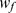, and natural myofiber strain during isovolumic contraction 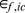, during ejection 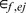, and during isovolumic relaxation 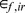. The 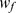 at each point is defined as the area enclosed by the myofiber Cauchy stress-natural strain loop:
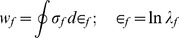
(5)Global cardiac function is quantified by maximum LV pressure 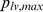 and stroke volume 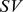.

#### Comparison with experimental data

Left ventricular torsion 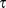 as computed with the model was compared with torsion determined from magnetic resonance tagging (MRT) experiments. The protocol for the MRT measurements and the definition of 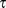 have been described previously in [6]. Essentially, 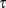 quantifies the base-to-apex gradient of rotation about the LV long axis (see figure 1). In the model, 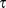 was determined at the levels of the MR slices by interpolation from adjacent nodal points in the FE mesh. In agreement with the experimental procedure, 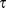 was computed with respect to begin-ejection and averaged in radial direction.
